# The effect of anti-coagulation dosage on the outcome of hospitalized COVID-19 patients in Ethiopia: a multi-center retrospective cohort study

**DOI:** 10.1186/s12890-023-02375-x

**Published:** 2023-03-13

**Authors:** Abel Girma Tessema, Zekarias Masresha Mengiste, Tsegaye Gebreyes Hundie, Hailemichael Getachew Yosef, Dawit Kebede Huluka, Abebaw Bekele Seyoum, Hannibal Kassahun Abate, Rawleigh Craig Howe

**Affiliations:** 1 Armauer Hansen Research Institute (AHRI), Addis Ababa, Ethiopia; 2 Eka-Kotebe General Hospital, Addis Ababa, Ethiopia; 3 Addis Ababa University, Addis Ababa, Ethiopia

**Keywords:** COVID-19, Thrombosis, Anticoagulation, Heparin, Enoxaparin, Bleeding, Ethiopia

## Abstract

**Background:**

Studies have indicated that hospitalized COVID-19 patients benefit from anticoagulation therapy in terms of survival; however, there is an ongoing controversy over the optimum anticoagulant dosage. This study aimed to compare clinical outcomes between patients who received prophylactic anticoagulation and those who received therapeutic anticoagulation.

**Methods:**

A multi-center retrospective cohort study was conducted to determine the impact of anticoagulation dosage in hospitalized COVID-19 patients in Ethiopia. The primary outcome measure was in-hospital mortality, and it was assessed using multivariable binary logistic regression and covariate-adjusted Cox Proportional Hazard model. For critical and severe COVID-19 patients, subgroup analyses were performed using multivariable binary logistic regression model and multivariable Cox regression models.

**Result:**

A total of 472 hospitalized COVID-19 patients were included in this study, of whom 235 (49.8%) received therapeutic anticoagulation and 237 (50.2%) received prophylactic dose. The demographic and baseline clinical characteristics were roughly similar between the groups. After adjustment for several confounders, in critical COVID-19 subgroup, therapeutic dose of anticoagulation was significantly associated with a higher inpatient mortality (AOR 2.27, 95% CI, 1.18—4.35, *p* = *0.013*), whereas in severe COVID-19 subgroup, anticoagulation dosage was not associated with inpatient mortality (OR, 1.02, 95% CI, 0.45 – 2.33, *p* = *0.958*). In severe COVID-19 patient group however, the incidence of thrombosis was slightly lower in the therapeutic group as compared with prophylactic group although the difference was not statistically significant (AOR 0.15, 95% CI, 0.02 – 1.20, *p* = *0.073*). Although there were only six major bleeding events in this study, all these were recorded from patients in the therapeutic subgroup, making the difference statistically significant (*p* = *0.013*).

**Conclusion:**

Although this study is limited by its observational design, our results are not consistent with current recommendations on anti-coagulation dose for hospitalized patients with COVID-19, necessitating the need for RCT in resource limited settings.

## Background

Several studies [1–5] have established that COVID-19 patients are at increased risk of developing thrombotic complications; however, the optimal dose of pharmaco-prophylaxis to administer to hospitalized patients is still a topic of controversy due to the ongoing emergence of conflicting evidence [6–9].

One of the earliest pieces of evidence on the benefit of anticoagulation for COVID-19 patients was from a retrospective study conducted in Tongji Hospital in Wuhan, China which showed that the 28-day mortality was significantly lower in heparin-users as compared to non-users [10]. This finding was further supported by other studies [11, 12], and as a result standard dose thromboprophylaxis was adopted as part of routine clinical care for COVID-19 patients who require inpatient care [7, 13]. Despite the use of low-dose anticoagulation however, in some studies [14], a high proportion of patients were still developing life-threatening thrombotic complications suggesting a need for more aggressive anticoagulation in hospitalized COVID-19 patients, especially in critical patients [15].

This prompted several observational and randomized controlled trial (RCT) studies comparing high-dose (therapeutic) anticoagulation and low-dose (prophylactic) anticoagulation in the outcome of hospitalized COVID-19 patients. Some studies came out supporting therapeutic anticoagulation while others revealed contradictory findings, indicating the possibility that administering high-dose anticoagulation without documented evidence of thrombosis provided no additional benefit [16–18].

The ACTION trial reported, in patients hospitalized with COVID-19 with elevated D-dimer concentration, therapeutic anticoagulation did not improve clinical outcomes and increased bleeding [6]. On the other end, the HEP-COVID RCT demonstrated significant reduction in thrombosis and mortality in patient who were provided with high-dose anticoagulation [8]. The finding of this trial was partly supported by a recent meta-analysis done on three randomized trials (REMAP-CAP, ACTIV-4a, and ATTACC), reporting superior outcomes in therapeutic patient groups for non-critical COVID-19 patients [19] and inferior outcomes for critical COVID-19 patients who received therapeutic anticoagulation as compared to those who received prophylactic anticoagulation [20, 21].

All this evidence and recommendations however are from studies done in high-income nations and require patient stratification based on laboratory tests like D-dimer, which are typically unavailable in most low- and middle-income settings. A pragmatic recommendation for low- and middle-income countries (LMICs) indicated administration of prophylactic anticoagulation for all hospitalized COVID-19 patients if there are no contraindications [7]. It recommended against empiric therapeutic anticoagulation in the absence of clinical suspicion for venous thromboembolism (VTE).

In the Ethiopian context, the current national COVID-19 treatment guideline, which was last updated in September 2020, recommends the use of therapeutic dose for critical and prophylactic dose for severe COVID-19 disease [13]. With the emergence of more recent evidence from RCTs recommending the opposite, there has been inconsistency in the dosage of anticoagulant usage in the country [19–21]. While some centers have changed the practice in accordance to the results of these RCTs, others are still adhering to the national guideline recommendation. As such, there is a need for a contextual study and update as there is no evidence on dosage and efficacy of anticoagulation, nor any studies on thrombotic complications of COVID-19 in Ethiopia. This study aims to provide insight into the effect of anticoagulation dose on clinical outcomes of hospitalized COVID-19 patients in Ethiopia.

## Methods

### Study design and setting

A health facility based retrospective multi-center cohort study comparing clinical outcomes in patients who received prophylactic anticoagulation and therapeutic anticoagulation was conducted by reviewing patients’ charts in the three largest COVID-19 treatment facilities in Addis Ababa, Ethiopia. The facilities included Eka-Kotebe General Hospital (EKGH), Millennium COVID-19 Care Center (MCCC) and Bulbula COVID-19 Field Hospital under St. Peter's Specialized Hospital (BFH). The data were obtained from the medical records of patients admitted at the study sites from April 15^th^ 2020 to January 15^th^ 2022.

### Sampling procedure and eligibility criteria

A study conducted in an Ethiopian hospital with a setting similar to ours reported a mortality rate of 28.6% among hospitalized COVID-19 patient [22]. Assuming a mortality rate of 25–30%, equal numbers of patients given therapeutic and prophylactic anticoagulation, a power of 0.8, and a 10–15% difference between the groups, we calculated a required sample size of 234–252 patients per group. Based on the total case load since the onset of COVID on each study site, a 2:2:1 proportion (EKGH: MCCC: BFH) was used to determine the sample size at each respective study site. In selection of the study patients, a simple random sampling technique was applied using the HMIS (Health Management Information System) at each study site as a sampling frame.

In all included patients, COVID-19 was confirmed by a positive polymerase chain reaction (PCR) test. In addition, all included patients were above 18 years of age by the time they were admitted to the hospital. On the day of hospital admission, all patients received either therapeutic or prophylactic dose pharmacological thromboprophylaxis, and the same regimen was followed for at least 7 days. The anticoagulation medications used were mainly unfractionated heparin (UFH) and the low molecular weight heparin (LMWH), enoxaparin. Other anticoagulants used were rivaroxaban and warfarin. The decision to put patients on which anticoagulant regimen was primarily based on local hospital guidelines and judgement of attending physicians, which varied frequently throughout the course of the period. We excluded patients who had any contraindication for anticoagulation, patients who were receiving anticoagulation therapy prior to the diagnosis of COVID-19 for other medical indication, patients who were diagnosed with thrombosis during or prior to hospitalization, or patients who did not have complete medical charts, including documented history, physical examination, investigation and management recorded throughout their hospital stay.

### Data collection and quality assurance

The data were collected using a pre-tested data collection tool guided by the reports of the ACTION randomized trial [6]. The tool was modified and commented by experienced pulmonologist and internal medicine specialists to ensure convenience in the setting. A pilot study was conducted on 25 patients from May 01, 2022—May 31, 2022, and modified appropriately including removal of unnecessary variables. The final data collection tool had four parts. The first part included demographics (age, gender, occupation) and hospital number. In the second part, patients’ baseline and admission characteristics were recorded, including variables such as vaccination status, presence of chronic co-morbid conditions (hypertension, diabetes, ischemic heart disease, heart failure, stroke, asthma, chronic kidney disease, chronic liver disease, HIV/AIDS, malignancies, and smoking), risk factors for thrombosis (based on the Well’s score), patients’ symptoms, physical findings, investigation results and management initiated at admission. In the third part, any significant changes that occurred during patients’ hospital stay including development of new symptoms, new physical findings, pertinent investigation findings, new diagnoses, new treatment initiated were recorded. In the final part, outcome of the patients and patients’ status on the day of the outcome were recorded. All-cause in-hospital mortality was the primary outcome variable, and length of hospital stay and thrombotic events were secondary outcome variables. As a safety outcome, we included bleeding complications.

In Ethiopia, different types of COVID-19 vaccines with differing schedules were administered, including Pfizer/BioNTech, AstraZeneca (Oxford), Janssen (Johnson & Johnson), and the Sinopharm vaccine. Although vaccination status was recorded upon admission and most patients specified the number of doses they took, they were mostly unaware of the specific vaccine type they received, making it challenging to determine the complete vaccination status of our study subjects. Hence, we resorted to categorizing patients as those who took at least one dose of vaccine and those who didn’t receive any.

Eight experienced and trained data collectors (all general practitioners working as clinicians in the study sites) collected the data from April 2022—July 2022. We utilized REDCap software for on-site electronic data collection, cleaning and management of the collected database. The data collectors were trained about the entire process of data collection including quality control measures such as: completeness, correctness, consistency, and synchronizing and archiving data with REDCap. Regular supervision and follow-up were made by the principal investigator throughout the data collection period. Completeness, correctness and consistency of the reviewed data were checked on a daily basis by supervisors. The overall activities and entire process of data collection were led by the principal investigator. The data collection process followed the standard national infection prevention and control protocol for COVID-19.

### Operational definitions

The definition and classification of COVID-19 was primarily based on the third edition of the national comprehensive COVID-19 clinical management handbook for Ethiopia with some modifications [13].

Confirmed COVID-19 case: A person with a laboratory confirmation of COVID-19 infection using a real-time polymerase chain reaction test (RT PCR test), irrespective of clinical signs and symptoms.

Severe COVID 19 illness: Confirmed COVID-19 patients receiving oxygen supplementation with either nasal cannula or simple face-mask but not requiring face-mask with reservoir.

Critical COVID 19 illness: Confirmed COVID-19 patients requiring oxygen supplementation with reservoir face-mask or mechanical ventilation or patients with respiratory failure, septic shock, and/or multiple organ dysfunctions (MOD) or failure (MOF) and needing invasive or special management.

Thrombotic complications (events): include the development of either arterial (acute myocardial infarction, acute limb ischemia, mesenteric ischemia, cerebral infarction, aortic thrombosis) or venous (pulmonary thromboembolism, deep vein thrombosis) thrombotic disorder [6]. Unless otherwise specified, thrombotic events refer to patients where the diagnosis of thrombosis was supported by definitive investigation modalities (i.e., definitive thrombosis).

Clinically diagnosed thrombosis: diagnosis of thrombotic complication evidenced by patient's clinical presentation but not necessarily confirmed by definitive investigation modality.

Definitively diagnosed thrombosis: diagnosis of thrombotic complication confirmed by definitive investigation modality.

Prophylactic anticoagulation dosage: include either of the following anticoagulation regimens:

- For patients with body mass index (BMI) < 40 kg/m2 → LMWH: (Enoxaparin 40 mg SC daily) or UFH 5000 IU SC (subcutaneously) TID (three times a day) or UFH 7500 IU SC BID (twice a day),
- For patients with BMI > 40 kg/m2 → Enoxaparin 40 mg SC BID,
- For patients with BMI ≥ 50 kg/m2 → Enoxaparin 60 mg SC BID and.
- For patients with creatinine clearance < 30 mL/min → Enoxaparin 30 mg SC daily.

Therapeutic anticoagulation dosage: include either of the following anticoagulation regimens:

- Enoxaparin 1 mg/kg SC BID (60 mg SC BID) for 45 days or UFH 5000 IU IV/SC bolus and then 17,500 IU SC BID until patient discharge with subsequent prescribed shift to oral anticoagulants, rivaroxaban 15 mg PO BID for 21 days, then 20 mg PO daily, or warfarin (three days overlap) dose adjusted to INR 2–3 for a total of 42 days.

Major or minor clinically significant bleeding [23].

(Adopted from International Society on Thrombosis and Hemostasis (ISTH) Definition).

Include one of the following: Fatal bleeding and/or Symptomatic bleeding in a critical area or organ, such as intracranial, intraspinal, intraocular, retroperitoneal, intraarticular or pericardial, or intramuscular with compartment syndrome or Bleeding causing a fall in hemoglobin level of 2 g/dL (1.24 mmol/L) or more, or leading to transfusion of two or more units of whole blood or red cells.

### Statistical analysis

All statistical analyses were performed using IBM Statistical Package for Social Sciences (SPSS) version 26 (IBM Corporation, Armonk, NY, USA). Continuous variables were analyzed using Mann Whitney U test and findings were reported as medians and interquartile ranges (IQR). Categorical variables were compared using Chi square for variables with high expected values and Fisher’s exact test for those with low expected values. Our primary outcome variable was all-cause in-hospital mortality. We used multivariable binary regression model to calculate the adjusted odds ratio. Additionally, a Cox proportional hazard model was used to see the effect of how different covariates impacted the time to death. Findings were reported as adjusted hazard ratio (AHR) with 95% confidence interval (CI). A Cox regression survival plot was also done stratified by COVID-19 severity at admission. Again, we used the Cox proportional hazard model to compare length of hospital stay, censoring patients who were transferred to another health facility and those who died in hospital. A p-value of less than 0.05 was considered statistically significant and all values were reported with a 95% CI.

## Results

### Socio-demographic and baseline clinical characteristics

In this study, a total of 472 patients diagnosed with COVID-19 were included. Among the study participants, 50.2% (237 patients) received a prophylactic dose of anticoagulation and 49.8% (235 patients) received a therapeutic dose of anticoagulation. The median age of the study subjects in the prophylactic and therapeutic groups was 59 years (IQR = 45.5 – 67.0) and 60 years (IQR = 48.0 – 67.0) respectively. There was a uniform distribution of underlying comorbidities in both groups with no statistically significant difference. The five commonest comorbidities included hypertension, diabetes, HIV/AIDS, asthma and stroke. Forty-six per cent (217 patients) required mechanical ventilation at some point during their hospital stay. In terms of COVID-19 severity, there was no statistically significant difference between the two cohort groups (*p* = *0.261*). In addition, there was no statistically significant difference in vital signs at presentation, with the exception of respiratory rate, which was higher for the therapeutic arm (*p* < *0.001*). Regarding baseline laboratory measurements, the only value which had statistically significant difference between the two groups was blood urea level [median = 32.50, IQR (21.3 – 46.5) in the prophylactic group vs [24.30, IQR (16.1 – 37.1) in the therapeutic group; *p* = *0.001*]. Table 1 summarizes the demographic and baseline clinical characteristics of the study participants.

**Table 1:** Demography, baseline characteristics and clinical variables of hospitalized COVID-19 patients in Ethiopia

		**Prophylactic anticoagulation (*n* = 237)**	**Therapeutic anticoagulation (*n* = 235)**	***p*-value**
**Hospital**	Eka-Kotebe General Hospital	83 (35.0%)	91 (38.7%)	0.648
	Millennium COVID-19 Care Center	99 (41.8%)	96 (40.9%)	
	COVID-19 St. Peter’s Field Hospital	55 (23.2%)	48 (20.4%)	
**Age in years, median (IQR)**		59.00 (45.5 – 67.0)	60.00 (48.0 – 67.0)	0.711
**Gender**	Male, n (%)	154 (65.0%)	135 (57.4%)	0.108
	Female, n (%)	83 (35.0%)	100 (42.6%)	
**Comorbidity, n (%)**				
Hypertension		85 (35.9%)	83 (35.3%)	0.924
Diabetes Mellitus		76 (32.1%)	74 (31.5%)	0.921
HIV/AIDS		11 (4.6%)	12 (5.1%)	0.834
Asthma		10 (4.2%)	13 (5.5%)	0.508
Stroke		7 (3.0%)	5 (2.1%)	0.772
Malignancy		2 (0.84%)	5 (2.1%)	0.284
Ischemic Heart Disease		3 (1.3%)	4 (1.7%)	0.724
Prior history of Tuberculosis		2 (0.8%)	5 (2.1%)	0.284
Chronic Kidney Disease		4 (1.69%)	1 (0.43%)	0.372
Chronic Liver Disease		2 (0.8%)	1 (0.43%)	1.000
Schizophrenia		0 (0.0%)	3 (1.3%)	0.123
**Smoking, n (%)**		3 (1.3%)	6 (2.6%)	0.337
**COVID-19 Severity at admission, n (%)**				
Severe		147 (62.0%)	133 (56.6%)	0.261
Critical		90 (38.0%)	102 (43.4%)	
**Type of Oxygen therapy used at admission, n (%)**				0.021
Nasal cannula		107 (45.1%)	80 (34.0%)	
Simple Face mask		40 (16.9%)	53 (22.6%)	
Face mask with reservoir		28 (11.8%)	41 (17.4%)	
Non-invasive ventilation		53 (22.4%)	44 (18.7%)	
Invasive ventilation		9 (3.8%)	17 (7.2%)	
**Vital Sign, median (IQR)**				
Heart rate, bpm		94.00 (86.0 – 106.0)	94.00 (83.0 – 105.0)	0.221
Respiratory rate, rate/min		28.00 (24.0 – 32.0)	31.00 (26.0 – 36.0)	< 0.001
Diastolic blood pressure, mm Hg		75.00 (67.0 – 83.5)	72.00 (65.0 – 80.0)	0.095
Systolic blood pressure, mm Hg		126.00 (111.5 – 145.0)	129.00 (112.0 – 142.0)	0.958
**Laboratory values, median (IQR)**				
Hemoglobin, g/dL		14.10 (13.0 – 15.5)	14.05 (13.0 – 15.3)	0881
Hematocrit, %		41.60 (38.4 – 45.2)	42.00 (38.5 – 45.0)	0.553
Total WBC count, × 10^3^ /mL		9.60 (6.4 – 11.9)	8.94 (6.4 – 13.1)	0.831
Neutrophil, %		87.50 (81.0 – 92.0)	89.85 (81.5 – 92.4)	0.144
Lymphocyte, %		6.35 (3.7 – 10.4)	6.30 (3.9 – 10.6)	0.745
Platelet count, 10^9^/L		244.50 (175.8 – 315.0)	234.00 (167.5 – 310.8)	0.622
Alanine aminotransferase, U/L		40.60 (25.6 – 65.1)	31.95 (22.8 – 62.5)	0.479
Aspartate aminotransferase, U/L		40.95 (29.1 – 62.2)	38.00 (27.1 – 60.1)	0.887
Creatinine, mg/dL		0.72 (0.62 – 0.90)	0.80 (0.60 – 1.0)	0.112
Urea, mg/dL		32.50 (21.3 – 46.5)	24.30 (16.1 – 37.1)	0.001
Sodium, mmol/L		138.00 (134.0 – 140.0)	137.00 (133.0 – 140.0)	0.456
Potassium, mmol/L		4.26 (3.90 – 4.70)	4.10 (3.70 – 4.60)	0.055

### In-hospital mortality in hospitalized COVID-19 patients

Among the 472 patients included in this study, 154 (32.6%) of them died during their hospital stay. All-cause in-hospital mortality was higher in the therapeutic cohort (37%, *n* = 88) as compared to the prophylactic cohort (28%, *n* = 66) (RR 1.35, 95% CI, 1.03—1.75, *p* = *0.027*).

We used a multivariable binary logistic regression model to control for the contribution of several clinical and demographic variables. Among these, those which showed an association with in-hospital mortality (*p* < *0.25*) in a univariate analysis were older age (> 60 years), not receiving at least one dose of COVID-19 vaccine, presence of hypertension, history of stroke, presence of malignancy, a higher level of severity of COVID-19 at admission and receiving therapeutic dosage of anticoagulation. After adjusting for these potential confounding variables, therapeutic dose of anticoagulation was still significantly associated with a higher inpatient mortality (AOR 1.70, 95% CI, 1.02 – 2.84, *p* = *0.042*). Other factors which remained significantly associated with a higher inpatient mortality after multivariable logistic regression analysis included: older age (> 60 years), not receiving at least one dose of COVID-19 vaccine, presence of malignancy, and a higher level of COVID-19 severity (*p* < *0.05*). Table 2 below displays the outcome of the binary logistic regression model with odds ratio for in-hospital mortality before and after adjustment for potential confounders (only variables with *p* < *0.25* in univariate analysis are shown in the table).

**Table 2:** Binary logistic regression analysis with odds ratio for in-hospital mortality

		**Inpatient mortality, n (%)**	**Total**	**Crude OR (95% CI)**	***p*-value**	**Adjusted OR (95% CI)**	***p*-value**
**Age**	< 60 yrs	66 (24.1%)	274				
	> 60 yrs	88 (44.4%)	198	2.52 (1.70–3.74)	< 0.001	2.74 (1.60–4.69)	< 0.001
**Vaccine status**	Not received	149 (33.7%)	442				
	Received at least 1 dose	5 (16.7%)	30	0.39 (0.15–1.05)	0.062	0.23 (0.07–0.84)	0.025
**Hypertension**	No	89 (27.4%)	304				
	Yes	65 (38.0%)	168	1.52 (1.03–2.27)	0.037	1.52 (0.87–2.65)	0.139
**History of Stroke**	No	148 (32.2%)	460				
	Yes	6 (50.0%)	12	2.11 (0.67–6.65)	0.203	2.44 (0.47–12.52)	0.286
**Malignancy**	No	148 (31.8%)	465				
	Yes	6 (85.7%)	7	12.85 (1.53–107.71)	0.019	19.70 (1.52–255.44)	0.023
**COVID-19 Severity**	Severe	25 (8.9%)	280				
	Critical	129 (67.2%)	192	20.56 (12.55–34.76)	< 0.001	23.37 (13.45–40.62)	< 0.001
**Anticoagulation dosage**	Prophylactic	66 (27.8%)	237				
	Therapeutic	88 (37.4%)	235	1.55 (1.05–2.29)	0.027	1.70 (1.02–2.84)	0.042

We did a subgroup analysis using multivariable binary logistic regression for in-hospital mortality to further account for the severity of COVID-19. In severe COVID-19 patients, there was no statistically significant difference between in-hospital mortality and anticoagulation dosage, where the odds of inpatient mortality in the therapeutic cohort was 1.02 (95% CI, 0.45 – 2.33); *p* = *0.958.* On the other hand, on the critical COVID-19 subgroup, therapeutic anticoagulation was significantly associated with a higher inpatient mortality (AOR 2.27, 95% CI, 1.18 – 4.35, *p* = *0.013*), when compared to prophylactic anticoagulation.

**Fig. 1:**
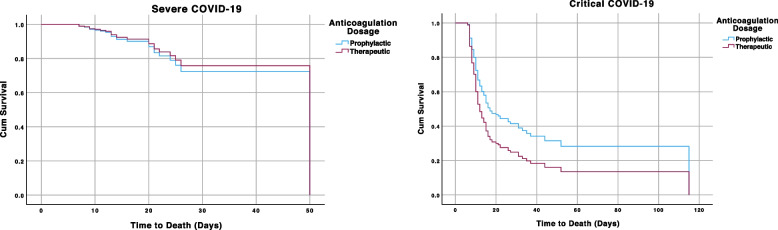
Cox proportional hazard cumulative survival plot. Left: survival plot severe COVID-19 patients receiving prophylactic vs therapeutic dose anticoagulation after adjusting for the listed confounders; Right: a survival plot for the critical subgroups

To further control confounders and see the effect of anticoagulation dosage on the time to death, we utilized a multivariable Cox regression analysis model. In the univariate Cox regression model, the potential predictors of mortality (*p* < *0.25*) included: older age, not receiving at least one dose of COVID-19 vaccine, hypertension, a higher level of severity of COVID-19 at admission, a higher demand of oxygen supplementation at admission and receiving therapeutic dosage of anticoagulation. After adjusting for these confounding variables, patients who received therapeutic dose of anticoagulation had significantly higher risk of death compared to prophylactically anticoagulated patients (AHR 1.41, 95% CI, 1.01 – 1.96, *p* = *0.042*) in multivariable Cox regression model. This effect was found to be more pronounced in a subgroup analysis done among critical COVID-19 patients (AHR 1.57, 95% CI, 1.09—2.27, *p* = *0.015*). However, there was no significant difference between the groups in the severe COVID-19 subset of patients, (HR 0.86, 95% CI 0.38—1.92, *p* = *0.711*). Cox regression analyses done for in-hospital mortality is summarized in Table 3 and Fig 1.

**Table 3:** Cox proportional hazard model for in-hospital mortality

	**Crude HR (95% CI)**^**a**^	***p*-value**	**Adjusted HR (95% CI)**^**a**^	***p*-value**
Age^b^	1.03 (1.02—1.04)	< 0.001	1.03 (1.02—1.04)	< 0.001
Vaccination Status	0.52 (0.21—1.26)	0.145	0.43 (0.17—1.06)	0.067
Hypertension	1.48 (1.07 – 2.04)	0.017	1.25 (0.90—1.75)	0.188
COVID-19 Severity	6.63 (4.31 – 10.10)	< 0.001	9.03 (4.30 – 18.99)	< 0.001
Anticoagulation dosage	1.23 (0.89 – 1.69)	0.215	1.41 (1.01 – 1.96)	0.042

^a^Hazard ratios of in-hospital mortality before and after adjusting for potential confounders given (only variables with *p* < 0.25 in univariate analysis are included)

^b^All independent variables are categorical except age which is continuous

### Length of hospital stay among survivors

Among the 472 patients enrolled in this study, 300 patients (63.6%) were discharged improved, 154 patients (32.6%) were deceased in the hospital and the remaining 18 patients (3.8%) were transferred to another health facility. To evaluate the length of stay among survivors, we again used a multivariable Cox regression analysis model, censoring deceased patients and those transferred to another health facility.

On univariate Cox regression analysis, variables associated with a longer hospital stay (*p* < *0.25*) included: older age, male sex, not receiving at least one dose of COVID-19 vaccine, history of stroke, a higher admission respiratory rate, a higher level of COVID-19 severity at admission, a higher oxygen demand at admission, and receiving therapeutic dosage of anticoagulation. After correction for these variables, the multivariable Cox regression analysis model indicated that therapeutic dose of anticoagulation was independently associated with a longer hospital stay as compared to prophylactic dose of anticoagulation (AHR 0.70, 95% CI, 0.55—0.91; *p* = *0.006*).

After stratification of patients based on COVID-19 severity, this effect was more prominent among the severe subgroup of patients (AHR 0.76, 95% CI, 0.59—0.98, *p* = *0.033*) and was lost for the critical subgroup (HR 0.61, 95% CI, 0.36 – 1.04, *p* = *0.069*). The following tables displays the Cox regression analysis summary of factors affecting the length of hospital stay in severe and critical COVID-19 patients (Table 4).

**Table 4:** Cox regression analysis for length of hospital stay

Covariates	Severe		Covariates	Critical	
	**Adjusted HR (95% CI)**	***p*-value**		**HR (95% CI)**	***p*-value**
**Anticoagulation dosage**	0.76 (0.59 – 0.98)	**0.033**	**Anticoagulation dosage**	0.61 (0.36 – 1.04)	**0.069**
**Age**	0.99 (0.98 – 1.00)	**0.047**			
**Gender (male sex)**	1.21 (0.93 – 1.58)	0.151			
**Stroke**	0.32 (0.12 – 0.88)	**0.027**			

Left: subgroup analysis for severe patients, Right: subgroup analysis for critical patients. (Variables with *p* < 0.25 in univariate analysis are included)

### Thrombotic and bleeding complications in hospitalized COVID-19 patients

Forty-two patients (8.90%) were clinically diagnosed with thrombosis during their hospital stay. Of these, 26 (62%) received a prophylactic dose of anticoagulation and 16 (38%) received a therapeutic dose. One patient was diagnosed with both pulmonary thrombo-embolism (PTE) and deep vein thrombosis (DVT). Two patients were diagnosed with both PTE and myocardial infarction (MI). One patient was diagnosed with PTE and acute limb ischemia. Confirmed diagnosis of thrombosis using definitive investigation modalities was achieved on only 13 patients (5.49%) of patients in the prophylactic group and 5 patients (2.13%) in the therapeutic group. The majority of these (two thirds) were venous thromboembolism (i.e., PTE and DVT). Therapeutic anticoagulation modestly decreased the incidence of definitive thrombosis in comparison with prophylactic anticoagulation (RR 0.39, 95% CI, 0.14—1.07, *p* = *0.07*); however, this association reached only borderline statistical significance. The difference in the incidence of thrombosis for severe subgroup of patients was relatively more pronounced (8 thrombosis in the prophylactic group vs. 1 in the therapeutic group; *p* = *0.038*), although this difference was lost in further multivariable binary logistic regression analysis (AOR 0.15, 95% CI, 0.02 – 1.20, *p* = *0.073*). However, this might have some clinical value despite it being statistically non-significant. For critical subgroup, 5 patients (2.1%) from the prophylactic cohort developed thrombotic events as compared to 4 patients (1.7%) from the therapeutic cohort (OR 0.69, 95% CI, 0.18 – 2.67, *p* = *0.595*).

The overall proportion of patients with bleeding events were 2.97% (*n* = 14). Among these only 43% (*n* = 6) were major or minor and clinically significant which resulted in either temporary or permanent discontinuation of anticoagulant. Comparing the two groups, patients who received therapeutic dose of anticoagulation had significantly higher bleeding risk as compared to prophylactic dose of anticoagulation [6 patients vs. 0 patient; *p* = *0.015*].

## Discussion

This multi-center retrospective cohort study compared the effects of prophylactic anticoagulation with therapeutic anticoagulation on the clinical outcomes of hospitalized COVID-19 patients with varying degrees of disease severity. Our key observations include: [1] The use of therapeutic dosage of anticoagulation did not improve survival among hospitalized COVID-19 patients; in fact, it may even have increased the risk of mortality, particularly among critical COVID-19 patients; [2] Severe COVID-19 patients who received therapeutic dose of anticoagulation had longer hospital stay than those who received prophylactic dose; [3] The incidence of thrombosis did not differ significantly between the therapeutic and prophylactic cohort groups, although in severe COVID-19 subgroup of patients therapeutic anticoagulation showed some protection against thrombosis (despite it being not statistically significant); [4] Patients who received therapeutic anticoagulation had significantly higher risk of major bleeding than those who received prophylactic anticoagulation.

There are several possible explanations for the higher in-hospital mortality observed among critical COVID-19 patients who received therapeutic dose of anticoagulation in our study. Compared to the prophylactic cohort group, the therapeutic cohort patients had a higher baseline respiratory rate, a higher demand for oxygen supplementation and a longer hospital stay, all of which may be indicative of more severe disease and may explain why the therapeutic groups had a higher inpatient mortality. Furthermore, since the decision to put which patients on high-dose anticoagulation was largely based on the clinical judgment of treating physicians, patients with more severe disease may be more likely to be put on higher dose of anticoagulation. We attempted to control for all these differences using multivariable models that took into account various variables that could reflect disease severity; however, we cannot rule out the possibility of an unaccounted for variable which could explain the differences in outcome apart from anti-coagulation dosage. Another obvious contributing factor is the occurrence of major bleeding in those patients who were provided with a therapeutic dose of anticoagulation. Although the number of bleeding events was small in this study, there was in fact a significant difference in the incidence of major bleeding between the two cohort groups. However, this cannot entirely explain the observed difference in mortality. Because of the restricted diagnostic capacity in all of our study centers, some bleedings may have been missed, possibly rendering the observed bleeding events in our study an under-representative. More studies—preferably randomized control trials—with larger sample sizes so that increased numbers of bleeding events can be identified, are required to substantiate this.

The clinical outcomes which were impacted by anticoagulation dosage were notably dependent on the level of COVID-19 severity at baseline. Among our critical COVID-19 patients (patients who required at least face-mask with reservoir at admission), our study indicates that there was clearly a higher prevalence of in-hospital mortality in patients who were in the therapeutic group. There was also a higher incidence of major bleeding events among these groups. Moreover, there was no significant difference in the incidence of thrombosis between the two cohort groups. Although the explanation for the occurrence of higher mortality could be any of those mentioned above, these finding make us question the use of therapeutic dosage in critical COVID-19 patients. On the other hand, on our severe COVID-19 sub-group of patients, there was no significant difference in the prevalence of in-hospital mortality between our two cohort groups. However, this finding may be impacted by the overall lower mortality in severe groups and might require a higher sample size to detect a difference. In terms of thrombotic events, in the severe COVID-19 subgroup, there was a lower incidence of proven thrombosis among patients who received therapeutic anticoagulation (8/237 in prophylactic vs 1/235 in therapeutic; AOR 0.15, 95% CI, 0.02 – 1.20, *p* = *0.073*), although this difference is not statistically significant. This finding, however, is severely limited by the paucity of diagnostic modalities to confirm suspected thrombosis, which considerably underrepresents the overall incidence of thrombotic events. In addition, the small number of events detected in this study might require a bigger study to further substantiate or refute this finding. Despite this however, among severe patients, therapeutic anticoagulation resulted in a significantly longer hospital stay and a slightly higher risk of bleeding as compared to prophylactic anticoagulation. This raises a concern whether the benefit of therapeutic doses of anticoagulation in severe COVID-19 patients subgroup outweighs the risk of longer hospital stay and higher bleeding events.

Our findings are consistent with a similar retrospective cohort study conducted on 311 COVID-19 patients admitted to Stony Brook University Hospital, which reported a possibility of higher inpatient mortality with the usage of high-dose anticoagulation. However, their study subjects included exclusively critical patients, and the majority of them were White population, which varied from ours [24]. Additionally, a large randomized clinical trial which included 615 patients concluded that the use of therapeutic anticoagulation should be avoided without evidence of other indication. They found no difference in their primary outcome measures (hierarchical analysis of time to death, duration of hospitalization, and duration of supplemental oxygen to day 30) between the groups, but found a significantly higher risk of bleeding with therapeutic anticoagulation. This study is however different from ours in it is a randomized clinical trial, included mainly moderate COVID-19 patients, the anticoagulant used was primarily rivaroxaban and the setting is in Brazil [6]. On the other hand, the analysis from the three RCTs (REMAP-CAP, ACTIV-4a, and ATTACC) found supporting evidence to the use of therapeutic anticoagulation particularly for non-critical COVID-19 patients and it was found to be non-beneficial for critical COVID-19 subgroup of patients [8, 9]. Our study found a similar result with these trials for the critical subgroup of patients where therapeutic anticoagulation had no survival benefit, but for the severe subgroup of patients (in their case “non-critical”), we found no statistically significant survival advantage unlike the report of the trials. This different result in the severe COVID-19 subgroup may be due to smaller sample size of our study, the difference in the study population (where our definition of severe COVID-19 included relatively more severe patients as compared to their “non-critical” patients), the difference in setting, and the difference in the study design. Although our study is merely an observational one, it is pertinent to our context because there is no available research on this controversial topic in Africa. The evidence we found from our data for critical COVID-19 patients in particular is in contrary to the recommendation of the current national COVID-19 treatment guideline. Although the practice has been changed in some centers following the result of recent RCTs, there is still inconsistency in practice from one treatment facility to another, highlighting the need for special attention and more investigation into this subject. Therefore, in addition to its practical clinical value, the study contributes to the advancement of scientific knowledge in our country.

This study has several limitations. To begin with, as the study is observational by its nature, we cannot make cause and effect conclusions, despite our attempts to control for residual confounding. Moreover, misclassification bias in distributing and selecting covariates leads to incomplete correction for confounding. To minimize these, we employed multivariable models with several covariates; however, to maximize the control for residual variables and for making a definitive conclusion, a randomized control trial will be needed. Second, a lack of diagnostic modalities such as spiral chest CT-scan, D-dimer, and doppler ultrasonography in some treatment facilities causes a large proportion of thrombotic complications to be left suspected and, in some cases, treated empirically. This has had an effect on our thrombosis incidence results. Third, due to the retrospective nature of the study and the unavailability of viral stain confirmation studies, we weren’t able to account for the different viral strain types. Finally, the retrospective nature of the research design introduces unintentional patient selection bias, which is one of the reasons why randomized clinical trials are regarded so critically.

## Conclusion

Overall, our findings are consistent with the possibility, in critical COVID-19 patients, therapeutic anticoagulation may provide no survival benefit, pose a higher risk of in-hospital mortality, have no additional protection against thrombotic complications, and pose a greater bleeding risk when compared to prophylactic anticoagulation. In severe COVID-19 subgroup, patients who received therapeutic anticoagulation were observed to have a longer hospital stay and did not seem to have improved survival, although there was a slight protection against thrombosis observed as compared to prophylactic group. As a result of these findings, we conclude that the benefit of therapeutic anticoagulation for hospitalized COVID-19 patients in Ethiopia should be addressed in future studies, and we emphasize the critical need for a randomized control trial in our setup to substantiate our findings.

## Data Availability

The datasets used and/or analyzed during the current study will be available from the corresponding author on reasonable request.

## Abbreviations

AHR: Adjusted Hazard Ratio
AOR: Adjusted Odds Ratio
ARDS: Acute Respiratory Distress Syndrome
BFH: Bulbula COVID-19 Field Hospital under Saint Peter’s Specialized Hospital
BID: Twice a day
BMI: Body Mass Index
CI: Confidence Interval
COVID-19: Coronavirus Disease of 2019
DVT: Deep Venous Thrombosis
EKGH: Eka Kotebe General Hospital
HIV/AIDS: Human Immunodeficiency Virus / Acquired Immunodeficiency Syndrome
HR: Hazard Ratio
IQR: Interquartile Range
ISTH: International Society on Thrombosis and Hemostasis
LMICs: Low- and Middle-Income Countries
LMWH: Low Molecular Weight Heparin
MCCC: Millennium COVID-19 Care Center
MI: Myocardial Infarction
MOD: Multi-organ Dysfunction
MOF: Multi-organ Failure
PCR: Polymerase Chain Reaction
PTE: Pulmonary Thromboembolism
RCT: Randomized Controlled Trial
RR: Relative Risk
RT-PCR: Real time Polymerase Chain Reaction
SC: Subcutaneous
SIC: Sepsis Induced Coagulopathy
SPSS: Statistical Package for Social Sciences
TID: Three times per day
UFH: Unfractionated Heparin
VTE: Venous Thromboembolism
